# Microscopic testicular sperm extraction in 46, XY differences in sex development caused by 5-alpha reductase type 2 deficiency

**DOI:** 10.20945/2359-4292-2024-0223

**Published:** 2025-02-10

**Authors:** Joao Paulo Greco Cardoso, Berenice Bilharinho Mendonça, William Carlos Nahas, Marcello Antonio Signorelli Cocuzza

**Affiliations:** 1 Divisão de Urologia, Hospital das Clínicas, Faculdade de Medicina, Universidade de São Paulo, São Paulo, SP Brasil; 2 Disciplina de Endocrinologia, Faculdade de Medicina, Universidade de São Paulo, São Paulo, SP Brasil

## Abstract

The 46, XY differences in sex development (DSD) caused by 5-alpha reductase type 2 (5ARD2) often presents with bilateral undescended testicles, otherwise normal internal reproductive structures, prostate hypoplasia and undervirilized male genitalia. Notably, as one of the few DSDs where fertility is possible, the clinical presentation of this disease is diverse, and reported cases of assisted reproduction are scarce. The fertility potential, reproductive counseling and treatment depend on the clinical presentation of this DSD, especially the testicular position and urethral anatomy. The influence of the timing and modality of surgery for hypospadias and cryptorchidism should be considered. We aimed to describe the use of microscopic testicular sperm extraction (micro-TESE) in this population. We provide a descriptive analysis of how micro-TESE is a possible potential tool for assisted reproduction in 5ARD2-deficient patients. A 33-year-old male who underwent bilateral orchidopexy, phalloplasty, and urethroplasty at the age of 9 years presented successful sperm retrieval but failed embryo development after intracytoplasmic sperm injection. Testicular histology revealed late spermatogenic arrest. A 28-year-old male with bilateral orchidopexy, phalloplasty, and urethroplasty at age 25 with unsuccessful sperm retrieval. Testicular histology revealed a Sertoli cell-only pattern. 5ARD2-deficient patients are singular patients. The potential impact of the time between atypical genitalia procedures and orchidopexy on fertility should be highly considered. Micro-TESE is a technique that may be used to assist azoospermic patients in this population. Early orchidopexy and penile and urethral corrections should be considered key strategies to preserve the fertility potential of 5ARD2 patients.

## INTRODUCTION

The 46, XY differences in sex development (DSD) due to deficient conversion of testosterone into its most active metabolite, dihydrotestosterone (DHT), is caused by 5-alpha reductase type 2 (5ARD2) deficiency (^1^). Defects in the 5ARD2 enzyme arise from mutations in the *SRD5A2* gene in either homozygous or compound heterozygous allelic variants. More than 129 allelic variations have been described. Variability in the molecular basis and potential differences in enzyme activity contribute to extensive phenotypic variability, even among individuals harboring the same mutation. 5ARD2 deficiency is a rare but globally distributed disease, with 434 reported cases across 44 countries (^2^).

At birth, 46,XY individuals present with several degrees of undervirilization of the external genitalia, ranging from typical female external genitalia to hypospadias or an isolated micropenis, frequent cryptorchidism, otherwise normal internal reproductive structures and prostate hypoplasia. The inability to convert testosterone into dihydrotestosterone affects the development of the male external genitalia (penis, penile urethra and scrotum) and the prostate. Structures derived from the mesonephric ducts (Wolff) are generally not affected since they are independent of DHT: the deferens, seminal vesicles, epididymis and ejaculatory ducts. Paramesonephric duct (Müller)-derived structures also do not evolve as they do in individuals without 46,XY; hence, their regression depends on the anti-Müllerian hormone produced by Sertoli cells. The association with cryptorchidism is prevalent, notwithstanding the uncertain role of dihydrotestosterone (DHT) in testicular migration. Clinically, 5ARD2 deficiency is suspected from atypical genitalia at birth and is confirmed by karyotype and genetic molecular diagnosis.

The 5ARD2 deficiency is among the rare causes of 46,XY differences in sex development (DSD), where paternity remains feasible, despite few cases being previously documented (^3-7^). The impairment of reproductive function is multifactorial. The anatomical variances in the external genitalia and urethra, even after corrective surgeries, may adversely affect sexual intercourse and ejaculation. Prostate hypoplasia, a prominent characteristic in most patients, markedly alters semen viscosity, volume, and ejaculatory dynamics. 5ARD2-deficient individuals usually present with a low seminal volume and high viscosity. The development of the testicular parenchyma is generally maintained; however, frequent association with cryptorchidism represents a potential source of gametogenesis impairment. Testosterone synthesis is typically preserved, maintaining the essential intratesticular levels necessary for spermatogenesis. Clinically, the hormonal profile often shows normal serum testosterone levels and lower levels of dihydrotestosterone (DHT), luteinizing hormone (LH), and follicle-stimulating hormone (FSH).

With respect to paternity outcomes, integrated and broad assistance to these populations should include these important aspects related to fertility, especially for patients who desire paternity (^8^). Ivarsson and cols. (^9^) reported two affected brothers in Sweden with spontaneous paternity. Katz and cols. (^3^) described the first assisted reproduction treatments through intrauterine injection on two successful occasions. Matsubara and cols. (^4^) reported the first successful intracytoplasmic sperm injection (ICSI) treatment, followed by Kang and cols. (^6^) and Costa and cols. (^10^) reports, also through ICSI. The latest report on fertility and paternity in 5ARD2 patients by Bertelloni and cols. (^5^). In 2019, two affected brothers, fathers through natural pregnancy and those conceived via ICSI assisted reproduction, were included.

This descriptive analysis provides the first reported use of testicular sperm extraction with microdissection (micro-TESE) in 5ARD2 patients. The study was approved by the Institutional Review Board Committee (*Comissão de Ética para Análise de Projetos de Pesquisa do Hospital das Clínicas Faculdade de Medicina da Universidade de São Paulo* [HCFMUSP] - board approval number 4.361.078) and was performed after written informed consent was obtained. The literature concerning previous cases of fertility and assisted reproduction in 5-alpha reductase type 2 deficiency patients was systematically searched across multiple electronic databases, including PubMed, Web of Science, Scopus, and Google Scholar, with the following keywords: “5-alpha reductase type 2 deficiency”; “Male infertility”; “Assisted reproduction”; and “Paternity”.

## MATERIALS AND METHODS

Two patients who were diagnosed with 5ARD2 deficiency were assisted at the Human Reproduction Center of the HCFMUSP, a tertiary and academic hospital.

*Patient 1:* Atypical genitalia diagnosed at birth and 5ARD2 deficiency established at age 9, after genetic evaluation: 46,XY karyotype and mutation analysis for *SRD5A2: p.G183S/p.G183S.* On the occasion, the patient had bilateral cryptorchidism with retractile testicles, perineal hypospadias with a single perineal opening, and a bifid scrotum. His penile length was 2.0 cm × 1 cm. Genital procedures were performed at age 9: orthophalloplasty with scrotoplasty, the first step of neourethroplasty and resection of the distal vagina with colpectomy. At age 10, the patient underwent a second step of neourethroplasty and bilateral orchidopexy. He came to the attention of the reproduction team at 36 years of age after a 5-year infertility history with his partner. They had weekly intercourse with satisfactory erections and/or movements but anejaculation. On physical examination, both testicles were located in the scrotum. No varicoceles were observed. Other features are presented in Table 1. Post-Ejaculate urine analysis was negative, excluding the possibility of retrograde ejaculation. The partner was evaluated by the gynecology team with an unremarkable history. The normal testicular size and hormonal profile with persistent azoospermia and negative test for retrograde ejaculation motivated the option for testicular sperm extraction via the microdissection (micro-TESE) technique.

**Table 1:** Clinical features of newly reported patients

Patient	Orchidopexy age	FSH (IU/L)	LH (IU/L)	T (ng/dL)	Testicular size	Histology	*SRD5A2* Mutation
Patient 1	9 yo	8.8	6.1	559	RT: 5 × 3 cm LT: 5 × 2.8 cm	Late maturation arrest	*p.Gli183Ser/p.Gli183Ser*
Patient 2	25 yo	19.9	11.4	918	RT: 4 × 2.8 cm LT: 3.5 × 2.4 cm	Sertoli cell-only	*p.Gln126Arg/p.Gln126Arg*

*Patient 2:* Atypical genitalia diagnosed at age 8 and 5ARD2 deficiency established at age 12, after genetic evaluation: 46,XY karyotype and mutation analysis for *SRD5A2: p.Q126R/p.Q126R.* At age 18, genital procedures had not yet been performed. He had bilateral cryptorchidism with an inguinal testicle, perineal hypospadias with a single perineal opening and a bifid scrotum. His penile length was 8.0 cm × 2.5 cm, and his testis size was 4.0 × 2.5 cm on the right and 3.5 × 2.4 cm on the left. Genital procedures were performed at ages 24 and 25: orthophalloplasty with urogenital sinus resection and closure followed by 2-step urethroplasty and orchidopexy. He came to the attention of the reproduction team at 29 years of age after a 10-year infertility history with his partner. They had weekly intercourse, and the patient described ejaculation in a small volume. The left testicle was still located in the inguinal canal and was 8 cc in volume. The right testicle was palpable in the scrotum and 8 cc in volume, and the Wolffian structures (epididymis and vas deferens) were apparently normal. No varicoceles were observed. Seminal analysis revealed a reduced volume of 0.2 mL, increased viscosity with extremely rare immotile sperm and a continuous positive Endtz test in multiple samples. The partner was evaluated by the gynecology team with an unremarkable history. Considering the inadequate number of seminal samples with extremely rare immotile sperm, we opted for testicular sperm extraction via the microdissection technique (micro-TESE).

Both patients and their partners were considered eligible for treatment. The ovarian reserve of the females was assessed through antral follicle counting and hormonal profiling. Tubal patency was assessed with hysterosalpingography. The male patients were assessed via testicular ultrasound, hormonal profiling, and genetic profiling. The selected treatment modality for both patients was micro-TESE, as previously described by Schlegel (^11^). Both testicular samples were analyzed during the surgical procedure and after the procedure in the embryology laboratory for the presence and selection of potential sperm for ICSI. For the definitive testicular biopsy, reminiscent tissue was analyzed after HE coloration under 100× and 400× optical magnification.

## RESULTS

**Figure 1:**
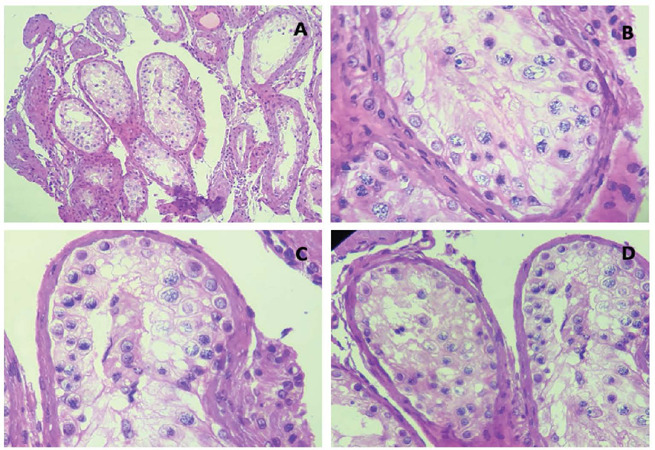
Patient 1 histology-late maturation arrest. H&E 100x (**A**) 400x (**B** to **D**)

*Patient 1:* Micro-TESE was performed in both testicles. The procedure was successful for sperm retrieval. Five oocytes in the M2 phase were injected with immotile sperm via the micro-TESE or ICSI technique. Three oocytes were potentially fertilized in the first 24 hours, according to the presence of 2 pronuclei. Unfortunately, there was no progression of cellular division in subsequent days. The final testicular biopsy report revealed tubular hyalinization and alterations in the basal membrane and germ cell population, with a late maturation arrest pattern, as demonstrated in Figure 1.

**Figure 2:**
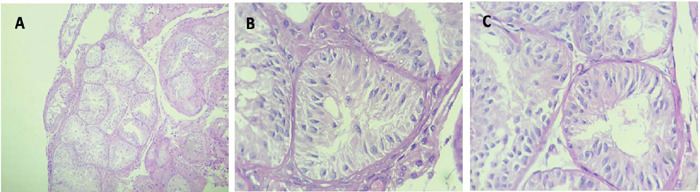
Patient 2 histology - Sertoli cell-only. H&E 100x (**A**) 400x (**B** to **D**)

*Patient 2:* Micro-TESE was performed in both testicles, including the left inguinal testicle. During the procedure, the microtubules were bilaterally atrophic and pale. The inguinal location of the left testicle represented a challenge to access. The procedure was unsuccessful in terms of sperm retrieval. The final testicular biopsy report revealed tubular hyalinization, alterations in the basal membrane and a Sertoli cell-only histological pattern, as demonstrated in Figure 2.

## DISCUSSION

Multiple aspects of 5ARD2 represent potential barriers to fertility (^8^), from gonadal development and placement to anatomical differences and previous surgical procedures. These aspects may alter spermatogenesis and semen dynamics to different degrees, reflecting the heterogeneous background of fertility status and paternity reports in the literature.

Considering the published data, summarized in Table 2, the patients with previous successful ART treatments had surgeries at birth (^5^), in a time frame of 9 to 17 years old (^4,10^), had spontaneous descent of testicles in puberty (^6^) or even had topical testicles at birth. With respect to testicular volume, patients with topical testicles had a greater mean testicular volume, 22.5 cc, with semen parameters that allowed 2 successful IUI treatments (^3^). The other patients presented with low sperm concentrations that demanded more invasive assisted reproduction through ICSI.

**Table 2:** Fertility and assisted reproduction in 5ARD2 patients

Reference	*SARD2* mutation	TP	MTV (cc)	LH, Ul/L	FSH, Ul/L	Tng/dL	DHT, ng/dL	T/DHT	Vol.	Cone. (106/mL)	Total (x 106)	M(%)	Mo	Age	Method	Pregnancy Outcome
1* Ivarsson (1996)	p.GIi196Ser/p.His231Arg	Palpable	...	...	...	917^a^	19^b^	48	...	...	...	...	...	23	Natural	Girl
2* Ivarsson (1996)	p.GIi196Ser/p.His231Arg	Not palpable	...	...	...	271^c^	...	...	...	...	...	...	...	22	Natural	Boy
**3 Katz and cols. (1997)**	p.Arg246Trp/p.Arg246Trp	Topic	22.5	...	...	669	9.8	68.2	0.5*	65	...	...	...	36 37	IUI 1 IUI 2	Boy Twins
**4 Matsubara and cols. (2010)**	p.Arg246Gln/Arg246Gln	Sx 9 yo	10.0	2.1	7.0	660	26	25	0.3	15	4.5	17	8	29	ICSI	Boy
**5 Kang and cols. (2011)**	p.Arg246Trp/p.Arg246Trp	Topic at puberty	15.0	...	...	669	23	29	<0.1&	8.4	...	<1.0	...	45	ICSI	Twins
6** Costa and cols. (2012)	p.Gln126Arg/p.Asn193Ser	Sx 14-17 yo	4.4	16.9	13.1	340	26	12	0.5	0.19	0.09	21	...	34	ICSI	Twins
7** Costa and cols. (2012)	p.Gln126Arg/p.Asn193Ser	Sx 15 yo	9.8	4.4	3.8	551	33.8	16	ND	#	...	...	...	30	ICSI	Boy
8*** Bertelloni and cols. (2019)	p.Arg103Pro/p.His230Pro	Sx at birth	12.5	6.4	25.8	320	...	...	...	...	...	...	...	32	Natural	2 Girls
9*** Bertelloni and cols. (2019)	p.Arg103Pro/p.His230Pro	Sx at birth	...	...	...	...	...	...	...	...	...				ICSI	Boy
**10 Present Report**	p.GIi183Ser/p.GIi183Ser	Sx 10 yo	15	6.1	8.8	559	6	93	∅	-	-	-	-	38	Failed ICSI after micro-TESE	-
**11 Present Report**	p.Gln126Arg/p.Gln126Arg	Sx25yo	8	11.4	19.9	980	...	...	0.2	§	-	-	-	29	Failed sperm retrieval micro-TESE	-

TP: testicular position: Sx (orchidopexy surgery) for patients 4, 6, 7, 8, 9, 10 and 11) and testicular position for patients 1, 2, 3 and 5. MTV: mean testicular volume: LH: luteinizing hormone: FSH: follicle-stimulating hormone: T: testosterone: DHT: dihydrotestosterone: T/DHT: testosterone dihydrotestosterone ratio: Mo: morphology: M: motility: Cone: concentration: ICSI: Intracytoplasmic sperm injection: ^a^at 14 yo: ^b^at 10 yo: ^c^at 36 yo. * Patients 1 and 2 brothers. ** Patients 6 and 7 brothers. *** Patients 6 and 7 brothers. ^&^ After centrifugation. ^#^ Rare motile sperm in the fresh ejaculate. 17 sperm selected for ICSI: ∅ anejaculation: negative post ejaculate urine analysis: ^§^ extremely rare immotile sperm

The use of ICSI is another important consideration in the advances of reproduction techniques that have allowed assistance at different levels of complexity. ICSI enables the treatment of azoospermic and oligozoospermic patients with their own gametes, including the use of testicular sperm (^12^).

In this series, orchidopexy and genitoplasty were performed at age 9 for Patient 1 and age 25 for Patient 2. The cases presented similar histopathological features, such as tubular hyalinization and alterations in the basal membrane and germ cell population. Nevertheless, the features of Patient 2 are more severe, with a Sertoli cell-only pattern that is reflected in unsuccessful sperm retrieval. Patient 1 presented a late maturation arrest pattern, with successful sperm retrieval, despite negative results after ICSI.

The time of the genital procedures and orchidopexy may have impacted the histological pattern. Although cryptorchidism is related to infertility, the consequences of prolonged testicular ectopy are not precisely defined. In our series, patient number 2, after 25 years of cryptorchidism, presented with a lower testicular volume, elevated FSH and LH levels and a histological pattern suggestive of testicular failure. Previous reports on the influence of time on the success of micro-TESE in this population are controversial. Raman and Schlegel (^13^) described a significant correlation between the sperm retrieval rate and orchidopexy age. Patients who underwent surgery before 10 years of age had higher rates of sperm retrieval. Wiser and cols. (^14^), however, did not reach the same conclusion regarding the timing of orchidopexy. In this study, a greater testicular volume was related to higher rates of testicular retrieval: 17.0 ± 6.3 mL in contrast with the 10.4 ± 6.3 mL (p = 0.05) of failed retrieval. The option for surgical sperm retrieval was distinct between patients 1 and 2, although both were based on previous success of the procedure, even in patients with cryptorchidism and testicular atrophy (^15^).

In conclusion, paternity desire and aspects related to fertility are major concerns in DSD patients, and the deficiency of 5ARD2 is one of the few 46,XY DSDs in which fertility is possible. In accordance with the scarce literature and the latest reported cases at this institution, early orchidopexy and genitoplasty should be considered strategies to prevent the impact of spermatogenesis and promote fertility preservation in this population. Despite these negative reported results, we would like to highlight the possibility of treating selected azoospermic 5ARD2 patients with micro-TESE.
